# High heat flux reduction to materials using current filaments

**DOI:** 10.1038/s41598-023-35109-4

**Published:** 2023-05-23

**Authors:** Trang Le, Yasuhiro Suzuki, Hiroki Hasegawa, Toseo Moritaka, Hiroaki Ohtani

**Affiliations:** 1 Institute of Research and Development, Duy Tan University, Da Nang, 550000 Vietnam; 2 Faculty of Natural Sciences, Duy Tan University, Da Nang, 550000 Vietnam; 3 Graduate School of Advanced Science and Engineering, Hiroshima University, Higashi-Hiroshima, 739-8527 Japan; 4 National Institute for Fusion Science, National Institutes of Natural Sciences, Toki, 509-5292 Japan; 5 The Graduate University for Advanced Studies, SOKENDAI, Toki, 509-5292 Japan

**Keywords:** Aerospace engineering, Astrophysical plasmas, Particle astrophysics

## Abstract

Reducing high electron and ion heat fluxes is one of the critical issues for shielding satellites and spacecraft. One of the ideas for shielding high particle and heat fluxes is to apply an external magnetic field generated by injecting current filaments. In this work, we model a flow of plasma, which includes electrons and ions in a small region, by using two spatial dimensions and three coordinates for velocities (2D3V) Particle-In-Cell (PIC) code to study the effects of the injected current filaments on particle and heat fluxes to the wall. The plasma enters the simulation domain from the source region at the left boundary and is fully absorbed in the conductor wall at the right boundary. Current filaments are injected to change the magnetic field structure of the system. We compare particle density, particle flux, and heat flux with and without injecting the current filaments into the domain in two dimensions. Based on the simulation results, we found that injecting current filaments can reduce the peak fluxes to the wall and transfer some of those fluxes along the wall. Therefore, injecting the current filaments is a good candidate for shielding satellites and spacecraft from high-energy ion and electron fluxes.

## Introduction

Plasma material interaction plays an important role in studying plasma physics in space and fusion plasma. In satellites or spacecraft, high energy ions may affect a single event upset and single event latch-up in space electronic systems, which causes software faults and can damage the device^1^. High-energy electrons can penetrate satellites and spacecraft and accumulate a charge on the surfaces of the conductor. A higher penetration rate causes internal charging and discharging pulses, which damage electronic systems or cause the failure of various spacecraft components^1–3^. Therefore, high-energy particles may damage material surfaces or deposit harmful charges into electronic components^4–6^. Shielding spacecraft or satellites from high-energy particles has become an important topic for space exploration. Several methods have been proposed to shield spacecraft and satellites from high-energy particles, such as active shielding methods, a chaotic magnetic field, or multi-layer shielding^7,8^.

On the other hand, the reduction of high-energy particles reaching the wall is also a significant issue in fusion engineering. High energy particles moving along magnetic field lines directly bombard the material, then damage end plates. Plasma detachment and resonance magnetic perturbations (RMPs) are some suggested solutions to reduce the high-energy plasma-wall interaction in fusion research. These methods are powerful techniques to reduce the high energy fluxes to the material, but they still have some remaining limitations related to technical challenges or physical issues^8–10^. Starting from studying high heat flux reduction in fusion plasma, we aimed to find a solution that can be applied for shielding spacecraft and satellites from high-energy particles.

One idea for high heat flux reduction is to expand plasma flow to match the width of the wall. Therefore, the plasma energy flux is spread over a larger area. This idea helps to reduce the burden of strongly localized fluxes on the material. It has been suggested that a magnetic field can affect the flux to the wall^11,12^. The magnetic field changes particle transport, which therefore influences the fluxes to the wall. In the previous work, we found that external localized-reversed magnetic fields can control the particle and heat fluxes to the wall in a one-dimensional view^13^. The particle and heat fluxes are reduced by the presence of magnetic mirror effects created by a localized-reversed magnetic field. There is a perspective of transferring the heat flux along the wall region. Therefore, we are curious to study how the flux profiles are affected along the wall region. This magnetic field profile can be generated by injecting the current filaments in experimental or two-dimensional (2D) numerical studies. To understand more qualitatively the effects of the external localized magnetic field, in other words, the current filaments, we study a flow of plasma consisting of electrons and ions in a small region by using two spatial dimensions and three coordinates for velocities (2D3V) Particle-In-Cell (PIC) model. PIC simulation is a model which uses a fully kinetic description to model the electrical potential structure self-consistently^14–16^. PIC uses micro-quantities to simulate all of the behaviors of plasma; therefore, it can deal with drifts explicitly compared to a fluid model^16^. We inject the current filaments in the direction which is perpendicular to the simulation plane. Localized plasma flow enters the simulation domain from the source region and is fully absorbed in the wall. This paper shows how injecting the current filament technique affects the particle and heat fluxes to the wall in two dimensions, which are the directions proportional to plasma flow and along the wall, using PIC simulation. Section “Simulation model” discusses how the simulation is set up, while section “Simulation results” compares density profiles, particle fluxes, and heat fluxes with and without using the current filaments. In the last section, the discussion and conclusion of this technique are given.

## Simulation model

In this work, we model the simulation using an electrostatic PIC model. The electric field **E** is self-consistently solved, and the magnetic field **B** is constant in time. The basic equations used in the present PIC simulation are similar to those in our previous work in a one-dimensional view^13^. Particle positions and field quantities are considered in two-dimensional *x* and *y* spaces with three components of velocity ($v_x, v_y,v_z$) (2D3V).

**Figure 1:**
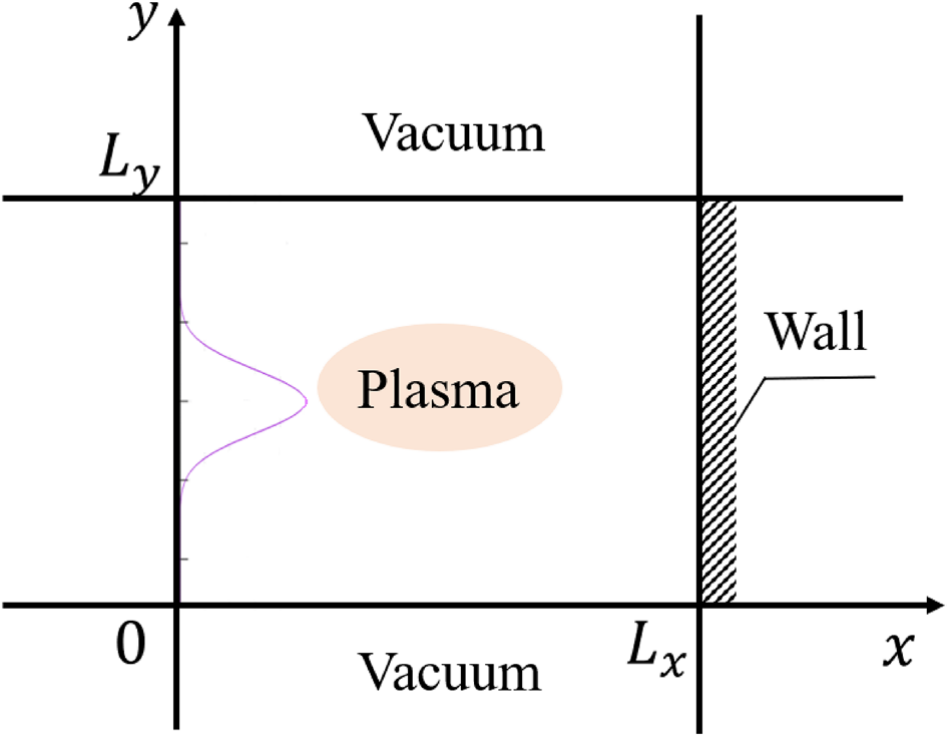
Simulation domain. The plasma is bounded by a vacuum. Particles are injected from the left boundary at $x = 0$ and be fully absorbed in the right boundary $x = L_x$. The purple line indicates the localized plasma flow entering the simulation domain.

We model a small, simple region where the plasma enters from the left boundary at $x = 0$ and is fully absorbed in the right boundary $x = L_x$. The simulation domain has the small size $L_x= 0.03$ m from the source to the conductor wall, and $L_y= 0.03$ m in the *y* direction. In the *y* direction, we assume that the plasma flows in the middle region of the simulation domain where the lower and upper boundaries are considered as a vacuum, as shown in Fig. 1. When a particle moves out of the simulation box from the upper and lower boundaries at the time *t*, it will be reflected into the simulation domain, given as $y_t^{refl}= 2L_y - y_t$ for $y_t>L_y$ or $y_t^{refl}= - y_t$ for $y_t<0$ and $v_t^{y,refl}= - v_t^y$. Only ions and electrons are included in this simulation. No collision, reflection, recycling process, or secondary emission has been considered.

**Figure 2:**
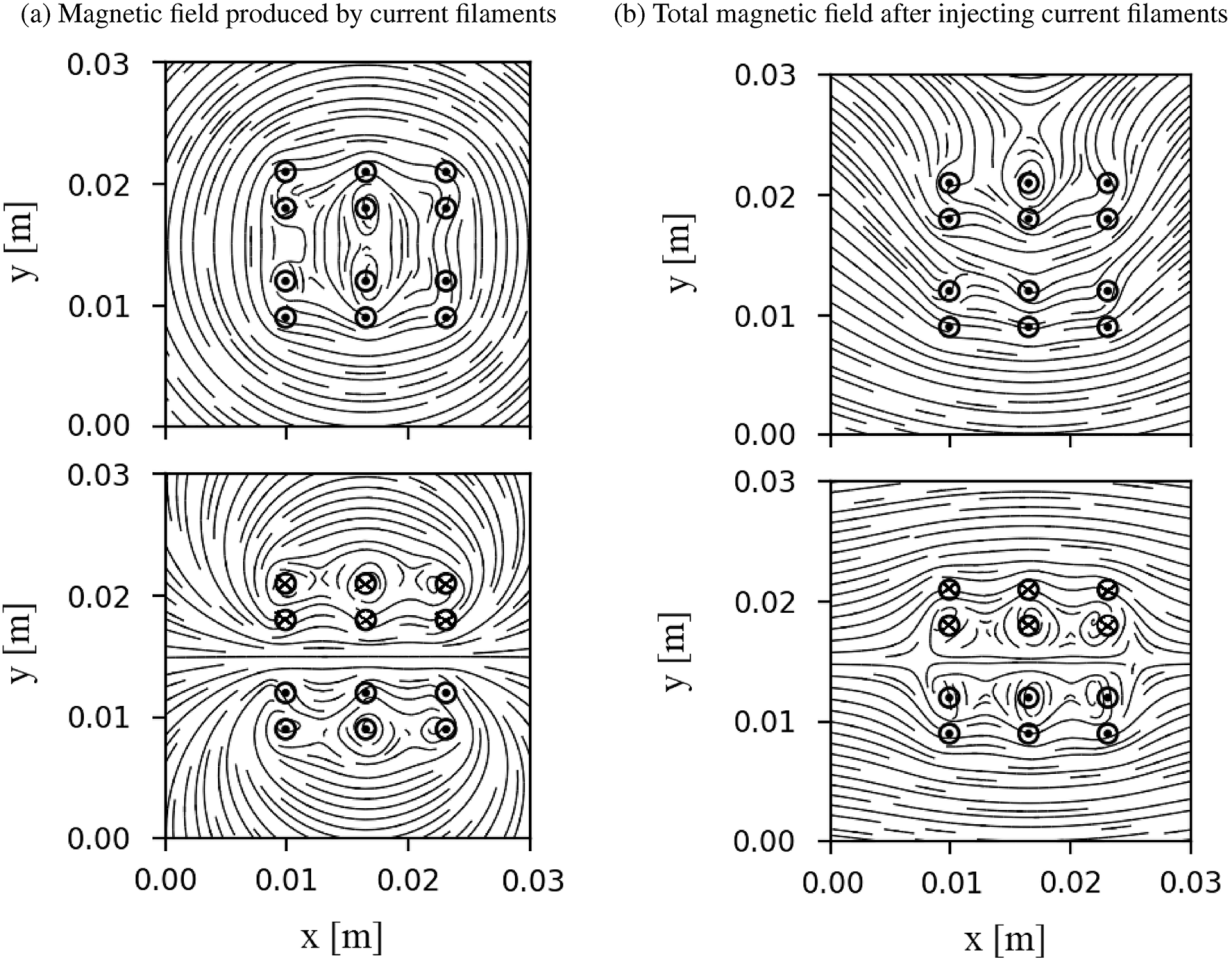
Magnetic field produced by the current filaments and total magnetic field in the simulation after injecting current filaments. Two types of injection are considered, including injecting the same current filaments and using opposite current filaments. The dot represents the outward direction, while the x denotes the inward direction of the current filament. The black line indicates the magnetic field line.

At the initial stage, there is no plasma inside the system. Particles are injected during each time step in the simulation, around $$x=0$$. We fix the particle flux at $$x=0$$, to be constant in time and have a Gaussian distribution profile, as indicated in the purple line in Fig. 1. This profile generates the localized distributions of the flux toward the wall targets. Electrons and ions have equal fluxes at the source $$x=0$$. The velocities of the injected ions and electrons follow a fully Maxwellian distribution function, which satisfies the condition that the parallel velocity $$v_{||} > 0$$. This work is an expansion of our one-dimensional view. Similar system parameters have been used to study heat flux reduction by the current filaments. The following parameters are used in the simulation: ion-electron mass ratio $$m_i/m_e=1836$$, electron source temperature $$T_{\textrm{e}0}=100$$ eV, ion source temperature $$T_{\textrm{i}0}=50$$ eV, background magnetic field $$B_x = 0.2 $$ T^13^. No magnetic field in the *y* and *z* directions are given. In the code, all of the parameters are normalized as:

1 $$\begin{aligned}{} & {} \hat{{\textbf {x}}} \leftarrow \frac{{\textbf {x}}}{\lambda _{De0}}, \hat{t} \leftarrow \omega _{pe0}t, \hat{v} \leftarrow \frac{v}{v_{e0}}, \hat{q} \leftarrow \frac{q}{e}, \hat{m} \leftarrow \frac{m}{m_e},\nonumber \\{} & {} \quad \hat{{\textbf {E}}} \leftarrow \frac{e {{\textbf {E}}}}{m_e \omega _{pe0}v_{e0}}, \hat{{\textbf {B}}} \leftarrow \frac{e {{\textbf {B}}}}{m_e \omega _{pe0}}, \hat{\rho } \leftarrow \frac{\rho }{n_0 e}, \hat{\phi } \leftarrow \frac{e \phi }{m_e v_{e0}^2}, \end{aligned}$$

where $\lambda _{De0} = \bigg ( \frac{\epsilon _0 kT_{e0}}{n_0 e^2} \bigg )^{1/2}$, $\omega _{pe0}=\bigg (\frac{n_0e^2}{\epsilon _0 m_e}\bigg )^{1/2}$, $\omega _{ce0}=\frac{e\textbf{B}}{m_e}$ and $v_{e0}$ are the Debye length, plasma frequency and cyclotron frequency, thermal velocity, respectively. *e*, $m_e$ and $n_0$ are the electric charge, mass and density of electrons, respectively. We choose $n_{0}=10^{16}$  $m^{-3}$ for normalization. With the above assumption, the simulation domain has the size $L_x=L_y \approx 40 \lambda _{De0}$. The system is started by setting the time step width $\Delta \hat{t}=0.02$ and the number of cells $N_{cell} = 300$ cells in each direction. The wall is assumed to satisfy a floating potential condition in which particles are fully absorbed. In the upper and lower boundaries (i.e. $y = 0$ and $y = L_y$) , the electric field is assumed to be equal to zero. Current filaments are injected into the system in the *z* direction, which is perpendicular to the system plane. The locations and directions of these filaments are given in Fig. 2. We perform the simulation in two cases: filaments have the same outward direction (Case 1), and filaments have the opposite direction (Case 2), corresponding to the upper and lower figures in Fig. 2a). In both cases, all filaments have the same current strength.The Biot–Savart law is used to compute the magnitude and direction of the magnetic field generated by a current given as:

2 $$\begin{aligned} B = \frac{\mu _0 I}{2 \pi r}= \frac{\mu _0 I}{2 \pi \sqrt{(x-x_{I})^2+(y-y_{I})^2}}, \end{aligned}$$

where $\mu _0=4\pi \times 10^{-7}$ Tm/A is the permeability of free space, $I=1$  kA is the current intensity and $x_{I}$, $y_{I}$ are the locations of the current filaments in the *x* and *y* directions, respectively. To avoid singularity of the magnetic field at the location of these injecting points, the magnetic field is cut down to $|B| = 1$ T. Injecting the current filaments changes the directions of magnetic field lines in the system. The magnetic structure in the simulation domain is different between these two cases. In the magnetized plasma, particles move along the magnetic field line. Because of these different structures, the transport of particles is different between these simulations, which will be discussed in the next section. Starting from $t =0$s, the simulation will terminate until the system reaches the equilibrium stage, in which all quantities will remain stable. We compare the particles’ quantities, such as particle flux with and without the injecting currents, to study the effects of the current filaments on the particle and heat fluxes to the wall.

## Simulation results

Defined $\Gamma _{s,\alpha }$ and $Q_{s,\alpha }$ are the particle and heat fluxes of the species *s* in the direction $\alpha$, given as

3 $$\begin{aligned}{} & {} \Gamma _{s,\alpha } = \int _{-\infty }^{\infty } dv_x \int _{-\infty }^{\infty } dv_y \int _{-\infty }^{\infty } dv_z v_{\alpha } f_s(\mathbf{{r,v}},t),\nonumber \\{} & {} \quad Q_{s,\alpha }=\int _{-\infty }^{\infty } dv_x \int _{-\infty }^{\infty } dv_y \int _{-\infty }^{\infty } dv_z \frac{m_s}{2}(v_x^2+v_y^2+v_z^2) v_{\alpha } f_s(\mathbf{{r,v}},t), \end{aligned}$$

where $f_s(\mathbf{{r,v}},t)$ is the particle distribution function at position $\textbf{r}$ and time *t* and $m_s$ is the mass of the particle of species *s*. In numerical calculations, these fluxes can be computed in a cell (*j*, *k*) at the location $(X_j,Y_k)$ as:

4 $$\begin{aligned}{} & {} \Gamma _{s,\alpha }(j,k) = \sum \limits _{i = 1}^{N_s} v_{s,\alpha ,i} S(X_j-\textrm{x}_{s,i}, Y_k-\textrm{y}_{s,i}),\nonumber \\{} & {} \quad Q_{s,\alpha }(j,k)= \sum \limits _{i = 1}^{N_s} \frac{m_s}{2}(v_{x,i}^2+v_{y,i}^2+v_{z,i}^2) v_{s,\alpha ,i} S(X_j-\textrm{x}_{s,i}, Y_k-\textrm{y}_{s,i}), \end{aligned}$$

where *S*(*x*, *y*) is the area weighting function and $N_s$ is the number of particles of the species *s* in the cell (*j*, *k*)^14^. The particle and heat fluxes are normalized as:

5 $$\begin{aligned} \hat{\Gamma } \leftarrow \frac{\Gamma }{v_{e0} n_0 } ,\quad \quad \quad \hat{Q} \leftarrow \frac{Q}{m_e v_{e0}^3 n_0}. \end{aligned}$$

The normalized particle flux $\hat{\Gamma }_{x}$ at $x=0$ is fixed to be constant with time and has the same value for electrons and ions, given by:

6 $$\begin{aligned} \hat{\Gamma }_{x}(y) = \hat{\Gamma }_{0} \exp (-\frac{(y-L_y/2\lambda _{De0})^2}{2}), \; \text { where} \; \hat{\Gamma }_{0} = 1.0; \end{aligned}$$

which has a strong peak value at the mid-region where $y = L_y/2$. Different values of $\hat{\Gamma }_{0}$ only affect the weight of particle fluxes entering the simulation box. They do not affect other quantities or movement of particles in the simulation domain. Therefore, the value of $\hat{\Gamma }_{0}$ does not affect the objective of this study, the effects of current filaments on particle and heat fluxes.

**Figure 3:**
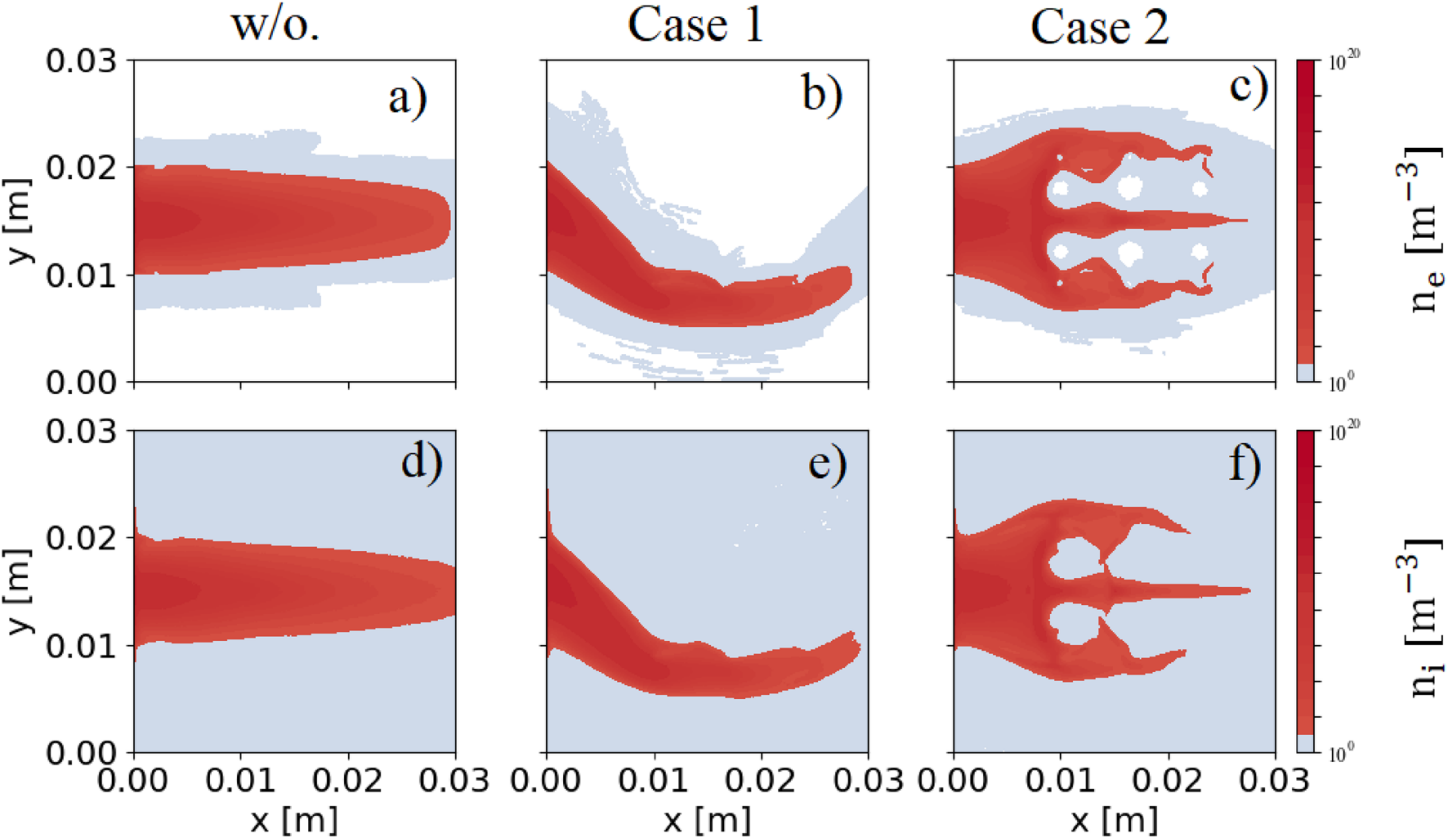
Particle densities at the equilibrium state with and without injecting the current filaments. Figures (**a**–**c**) are the electron densities, while Figs. (**d**–**f**) are ion densities. The flow of the particle transport depends on the total magnetic field structure. A sharpened profile is obtained by using the same direction of filaments (Case 1). The opposite direction of current filaments broadens particle flow in the simulation (Case 2).

**Figure 4:**
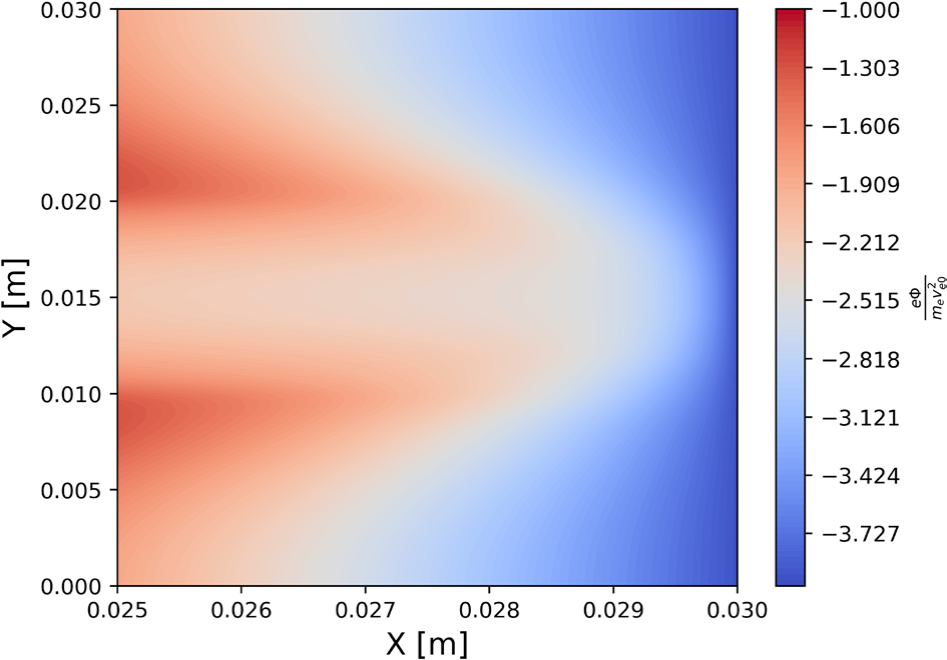
2D potential profile at the equilibrium state without injecting the current filaments. The figure is enlarged to show the area close to the wall. The formation of the sheath potential is confirmed.

Figure 3 shows the particle density in the simulation box. Without the external magnetic field, because of no magnetic field $B_y$ component, particles bombard the wall directly without changing its original shape (see Fig. 3a) for electron density and Fig. 3d for ion density). As shown in Figs. 3a and d, ion density in front of the target is higher than electron density. This unbalance comes from the formation of the sheath potential. To confirm the sheath potential formation, Fig. 4 shows a 2D profile of the plasma potential close to the wall at the equilibrium state without injecting the current filaments.

The sheath potential is self-consistently formed in front of the wall target to protect the losses of particles at the boundaries. Ions are accelerated to reach the wall, while most of electrons are reflected by the sheath potential. As a result, electrons are confined in the simulation box. More ions are located in front of the target than electrons. The sheath potential causes the energy exchange between electrons and ions. The energy of the ions at the target might drastically increase, which will affect the degradation of the plasma-facing components. Based on the self-consistently formation of the sheath potential in the case without injecting the current filaments, the code is verified to study the effect of the current filaments. Having a small Larmor radius, electrons mostly move along the field lines toward the wall. Electron flow follows the mainstream and rarely penetrates into a wider position in the *y* direction. Ions have a much larger Larmor radius. Ions having a large enough Larmor radius can jump to a large displacement in the *y* direction when composing the gyro orbit. Therefore, in the ion densities figures, we obtained low ion density in the region outside the mainstream, while electron density in this region is nearly equal to zero, which was indicated by the white color as shown in Fig. 3. When the current filaments are added to the simulation, the structure of the magnetic field is different as compared with without injecting current filaments. Subsequently, plasma flow is changed, corresponding to the change of the magnetic field line. Consider the first case when the same direction of the current filaments is used. In this case, as shown in the top figure of Fig. 2b), most of the magnetic field lines originated from the source are bending in the same direction around current filaments. They are convergent from the source toward the first current filaments’ location. The flow of particles entering the simulation box will be downwardly tightened in this region before moving further in the simulation box. Therefore, the flow of plasma is tightened toward the wall, as displayed in Figs. 3b and e for electron and ion densities, respectively. On the other hand, using the opposite direction of the filaments diverges the magnetic field lines from the source toward the first current filaments, as displayed in the bottom figure of Fig. 2 b). These divergent magnetic structures help to expand the localized plasma flow entering the simulation box in the larger area in the *y* direction, as shown in Figs. 3c and f. This expansion helps to decrease the number of particles reaching the wall and straighten out localized plasma flow entering the wall target.

**Figure 5:**
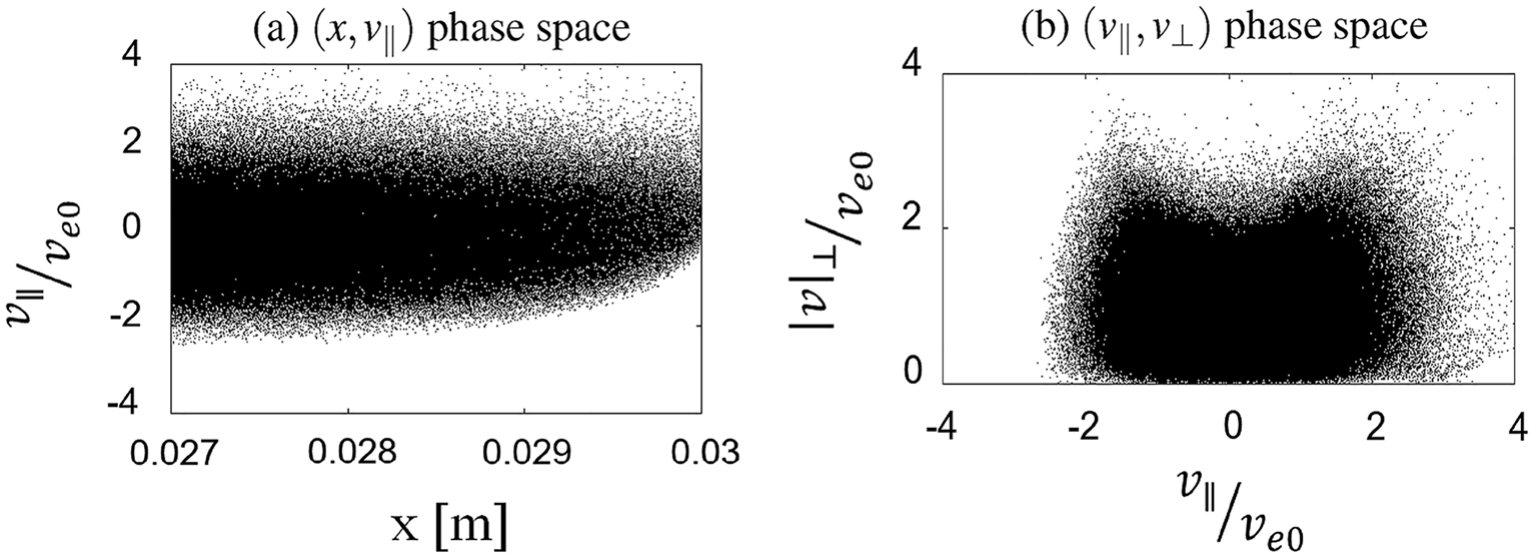
The electron distribution in (**a**) $(x, v_{\parallel })$ and (**b**) $(v_{\parallel },v_{\perp })$ phase spaces in the region near the wall target in the case of injecting the current filaments. Each dot represents each simulated particle.

The principle of trapping particles in the simulation box is based on the effect of the magnetic mirrors generated by injecting the current filaments. Due to the profile of the magnetic field produced by a current which is strong near the current and weaker in others, the magnetic mirrors are formed between the current filaments. From the source region where the magnetic field is weaker, particles move in the simulation region to meet the high field region. Particles are forced to reflect to the source region when entering these high field regions. Subsequently, the number of particles on the left side is higher than that on the right side of the current filaments. Fewer particles can be forced to move to the wall region. Particles are trapped by the magnetic mirror effects and expanded due to the divergence of magnetic field lines in the simulation region rather than moving toward the wall. The effect of magnetic mirrors has been shown in the velocity space figure. Figure 5 shows the electron distribution in (a) $(x, v_{\parallel })$ and (b) $(v_{\parallel },v_{\perp })$ phase spaces in the region near the wall target in the case of injecting the current filaments. The distance of the region corresponds to roughly $5 \times \lambda _{\text{D}}$. In the case there is no emission of the electron from the wall, the sheath potential repels the flow of the entering electrons. Only the particles with high parallel velocity can reach the target, as displayed in Fig. 5a. Due to the magnetic mirror, particles having high perpendicular and low parallel velocities hardly come into the wall region. They are trapped inside the magnetic mirrors. Only the particles having high parallel velocity can escape the magnetic mirrors to enter the wall region. As displayed in Fig. 5b, the loss cone with a bowl shape is obtained due to the magnetic mirrors. High perpendicular and nearly zero parallel velocity particles are prevented from reaching the target. Therefore, the particles are kept inside the simulation box rather than reaching the wall. The number of particles coming closer to the wall target is reduced by using the current filaments. Based on the different shapes of the particle flows reaching the wall, the particle and heat fluxes at the target are different in each case of the different direction of injection.

**Figure 6:**
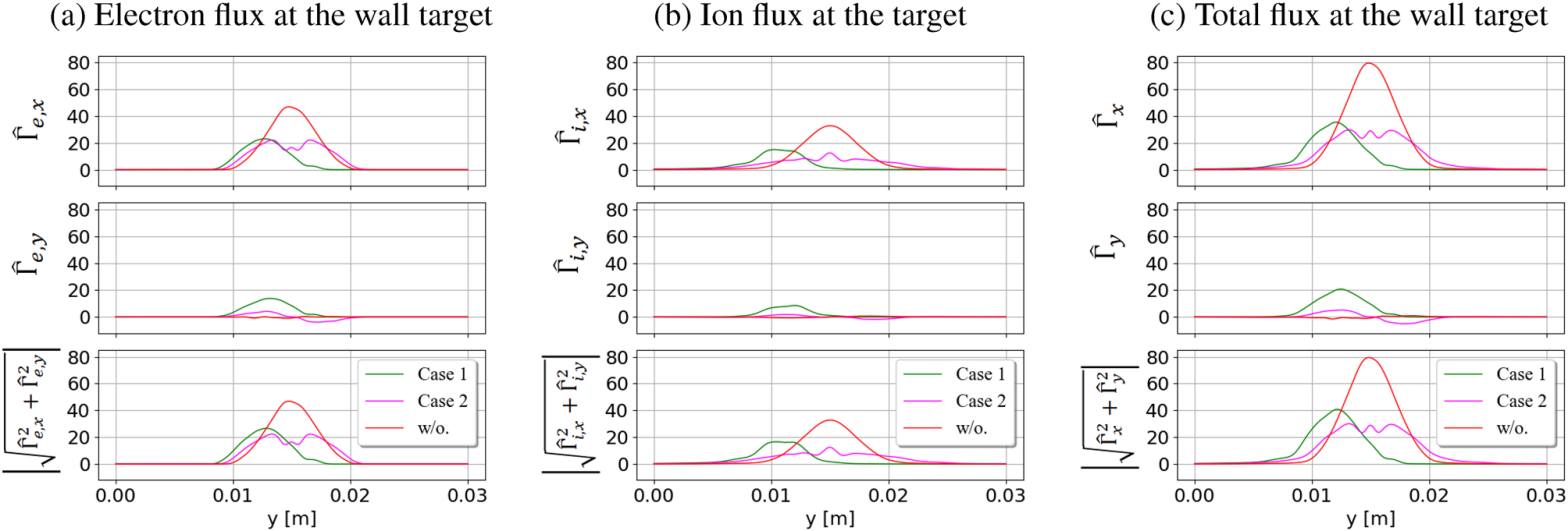
Electron, ion, and total particle fluxes at the wall target. Injecting the current filaments reduces electron flux in the *x* direction while changing the flux in the *y* direction. However, the total flux to the wall is reduced. Using the opposite direction of filaments (Case 2) can expand the flux along the target.

Figure 6 shows the fluxes of (**a**) electrons, (**b**) ions, and (**c**) total particles along the wall target, respectively. Without the external magnetic field, the localized fluxes entering the simulation domain result in the localized fluxes hitting the wall. These high fluxes are reduced by injecting the current filaments into the simulation system. The peak values of particle fluxes are reduced by half in the presence of the current filaments. As given in Eqs. (3) and (4), the particle flux is proportional to the density of particles. Once the particle density decreases, particle flux is also reduced. Due to the magnetic mirror effects formed between the current filaments, the particles are mostly trapped inside the simulation domain. The mirrors prevent a large number of particles from reaching the target. The density of particles near the wall region is decreased after injecting the current filaments, as shown in Fig. 3. Therefore, the particle flux at the target is reduced, corresponding to the drop in the particle density. In Case 1, the plasma flow is downwardly tightened toward the target due to bend magnetic field lines. The center of the localized distribution to the wall is shifted. Subsequently, on the flux figure at the target, the flux shows a shrink and shifted distribution as indicated in the green color in Fig. 6. On the other hand, the plasma flow is widely stretched out inside the simulation box by using the opposite direction of the current filaments, as in Case 2. In comparison with the case without injecting the current filaments, the plasma flow to the target is prolonged at the target. The particle flux at the target is expanded in Case 2, as pointed out by the magenta color in Fig. 6. The changing of plasma flow in the *y* direction comes from the magnetic field $B_y$ generated by the current filaments. The $B_y$ component may drive the particles moving in this direction. The particle flux transfers some of its flux to the *y* direction. The transferring process to other directions will help to reduce the strong flux to the target. The change of particle flux in the *y* direction can be seen in the wall target figures. Without injecting the current filaments, the particle flux $\Gamma _y$ at the wall target is almost equal to zero due to no collision and emission effects. In Case 1, because $B_y$, which is generated by the current filaments, has the same positive upward direction in front of the target, the particle flux $\Gamma _y$ is also same positive direction. In Case 2, $B_y$ near the wall has the opposite direction from the middle line $y=L_y/2$. The flux in the *y* direction is changed based on the direction of $B_y$, then is smaller in Case 2 than in Case 1. Particle flux in the *y* direction is much smaller than particle flux in the *x* direction. Even though injecting the current filaments reduces particle fluxes in the *x* direction and changes the flux in the *y* direction, the total particle flux at the target $\sqrt{\Gamma _x^2+\Gamma _y^2}$ is reduced in both cases under two-dimensional consideration. In conclusion, particle flux to the wall target is reduced by injecting the current filaments. The reduction of the particle flux mainly comes from the reflection process of particles caused by magnetic mirrors and the transfer of particle flux from the *x* direction to the *y* direction. Magnetic mirrors trap the particles inside the mirrors by forcing them to reflect back into the source region and allowing only particles having high parallel velocity can entering the wall. Due to this negative direction of reflected particles, in sum up, for all particles, the particle flux to the wall target is decreased.

**Figure 7:**
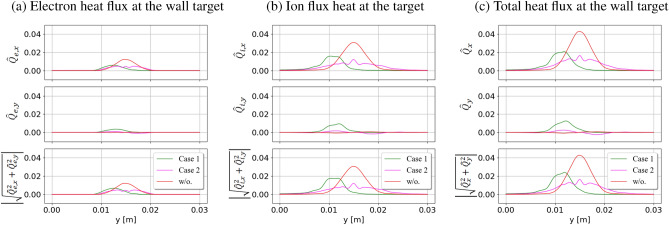
Electron, ion, and total heat fluxes at the wall target. Like particle flux, the heat flux to the wall is reduced by using the current filaments. Using the same direction of filaments (green line) sharpens the flux at the target, while injecting the opposite direction of the filaments (magenta line) expands the flux along the wall.

Similar behavior is obtained for the heat flux, as displayed in Fig. 7. Injecting the current filaments reduces high peak fluxes at the target. Using the same direction of the current filaments causes higher heat fluxes in the *y* direction and tightens the localized distribution of the fluxes. While the opposite direction of the current filaments helps to expand the fluxes along the target to reduce the peak burden for materials. If we want to concentrate on reducing the high peak fluxes to the target, injecting the same direction of current filaments into the system can be applied. Injecting the current filaments can be implemented in the experiment, for example, by using lower hybrid waves in the EAST tokamak^12^. For both flux reduction and expansion considerations, using the opposite direction of filaments is a better choice. Of course, this technique requires special treatment to change the direction of filaments when performing the experiments. Regarding experimental research, modern heat flux sensors are preferable to measure the heat flux in a high-temperature environment.

**Figure 8:**
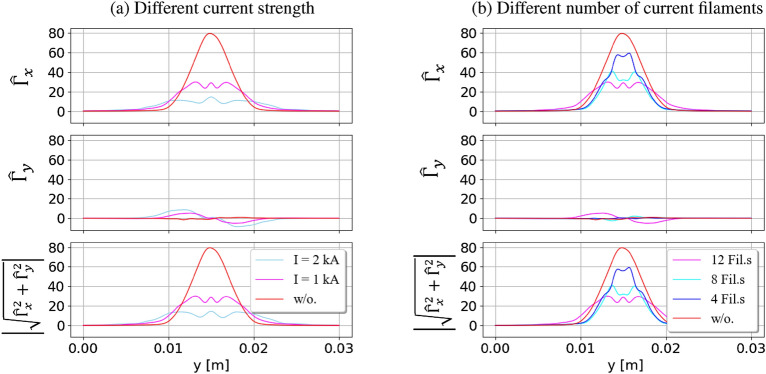
Comparison of total particle fluxes at the wall target by using different current strengths and different numbers of current filaments. The strength and number of current filaments affect the reduction of particle flux at the targets.

The magnetic field generated by the current filaments depends not only on their direction but also on their strength and number. Adjusting the number or the intensity of the current filaments may alter the magnetic field structure of the system, and influence the particle flux to the wall. Figure 8 illustrates the particle fluxes at the target by using (a) different current strengths and (b) different numbers of the current filaments. The opposite direction of the current filaments is used in this comparison. A stronger current is applied, and the change in the magnetic field becomes more obvious. The magnetic field in the *y* direction is stronger, driving more particles moving in the *y* direction. Therefore, a wider expansion of particle flow in the *y* direction occurs when using high current intensity. The localized plasma flow is stretched out in the simulation box before reaching the wall. A greater amount of particle flux, then, will be transferred into the *y* direction. This process will reduce the flux reaching the target. Consequently, a higher reduction and larger expansion of the particle flux at the target are obtained using strong filaments’ current intensity. Similarly, increasing the number of injected current filaments is proportional to reducing the particle flux at the target. A greater number of injected current filaments generates a larger number of magnetic mirrors in the simulation domain. More magnetic mirrors can trap a larger number of particles in the simulation domain. The number of particles reaching the wall target will be strongly reduced by having more magnetic mirrors. Therefore, the particle flux at the target will be significantly reduced by injecting more current filaments into the simulation, as shown in Fig. 8b. In total, the reduction and expansion of the particle flux at the target are dependent on the strength and number of the current filaments. Depending on the shape of the devices, the values of particle flux and magnetic configuration, the strength and number of current filaments should be considered to obtain high efficiency in the particle flux reduction to the targets in the experiments.

Once the simulation box is small, the distance from the source to the nearest current filament might be quite close. The magnetic field structure at the source is significantly changed compared to the case without injecting the current filaments, influencing the loaded particles into the simulation region from the left boundary. The injected particles are under both effects of the magnetic field change and the boundary conditions. To confirm the reduction of particle flux by using the current filaments, an expansion of the simulation box is considered. The system in which the length has been set as $L_x=L_y=0.05$ m is tested. The distance from the source to the nearest current filament is enlarged while the distance from the current filaments to the wall is the same as in the original simulation, $L_x=L_y=0.03$ m. By doing this, the magnetic field produced by the current filaments has a low impact on the injected particles at the source. Therefore, the particles can smoothly penetrate into the simulation domain. The opposite direction of the current filaments is used in this consideration. In the new simulation, the particle flow is similar to that discussed in the small simulation domain case. When using the current filaments, the particles bombard directly to the wall. The current filaments affect the shape of plasma flow. In this case, plasma flow near the source is expanded into a larger area in the *y* direction due to the opposite direction of the current filaments. Electrons and ions are trapped near the source region rather than reaching the wall. Compared with the case without injecting the current filaments, the total particle fluxes at the target are reduced by using the current filaments . In both different sizes of the simulation box, the injecting current filaments method still works well for reducing the intense particle flux to the target.

## Summary and discussion

This paper studies the effects of injecting current filaments on particle and heat fluxes using the 2D3V PIC simulation. We model a simple simulation, assuming that particles enter the simulation domain at $x=0$ and are fully absorbed in the wall. No collision or secondary emission is considered. Particles are injected from the left side with localized fluxes equal to ions and electrons and are constant with time. We compare the quantities of particles, such as particle flux, with and without injecting the currents. It can be concluded that current filaments change the magnetic structure in the system, then change the particle transport toward the wall. Particles return to the source region rather than reaching the wall target. Therefore, this method can decrease the number of particles reaching the wall and reduce high particle and heat fluxes to the wall. The reduction and expansion of the particle flux by injecting the current filaments depend on the strength and number of current filaments. Deeper reduction and larger expansion of the particle flux can be obtained by changing the strength and number of current filaments. To perform real experiments, different strengths and numbers of the current filaments will be used based on the wall boundary conditions or system input parameters. Using the same direction of current filaments reduces the peak of the fluxes at the wall while sharpening the localized fluxes. Using the opposite direction of current filaments, as proposed in this work, high peak fluxes to the wall can be reduced, and these localized fluxes can be expanded along the targets. In summary, injecting the current filaments is a good candidate to reduce the high heat fluxes to the target. This method can be applied to shield satellites or spacecrafts from high-energy particles. The effects of collisionality and finite current filaments have to be studied with PIC simulation in the future. From that perspective, additional boundary conditions are considered to make the simulation more realistic. That will be discussed in a different paper.

## Data Availability

The datasets used and/or analyzed during the current study are available from the corresponding author on reasonable request.
